# A Rapid, Stability Indicating RP-UPLC Method for Simultaneous Determination of Ambroxol Hydrochloride, Cetirizine Hydrochloride and Antimicrobial Preservatives in Liquid Pharmaceutical Formulation

**DOI:** 10.3797/scipharm.1103-19

**Published:** 2011-05-26

**Authors:** Rakshit Kanubhai Trivedi, Mukesh C. Patel, Sushant B. Jadhav

**Affiliations:** 1 Analytical Research and Development, Integrated Product Development, Dr. Reddy’s Laboratories Ltd., Bachupally, Hyderabad-500 072, India; 2 P.S. Science and H.D. Patel Arts College, S.V. Campus, Kadi-382 715, Gujarat, India

**Keywords:** Cetirizine dihydrochloride, Methylparaben, Propylparaben, *Levo*-cetirizine, Bromhexine, Method validation, Forced degradation, Oral solution, Assay, Chromatography, UV spectra

## Abstract

A stability indicating reversed phase ultra performance liquid chromatography (RP-UPLC) method was developed for simultaneous determination of ambroxol hydrochloride (AMB), cetirizine hydrochloride (CTZ), methylparaben (MP) and propylparaben (PP) in liquid pharmaceutical formulation. The desired chromatographic separation was achieved on an Agilent Eclipse plus C18, 1.8 μm (50 × 2.1 mm) column using gradient elution at 237 nm detector wavelength. The optimized mobile phase consists of a mixture of 0.01 M phosphate buffer and 0.1 % triethylamine as a solvent-A and acetonitrile as a solvent-B. The developed method separates AMB, CTZ, MP and PP in presence of twelve known impurities/degradation products and one unknown degradation product within 3.5 min. Stability indicating capability was established by forced degradation experiments and seperation of known and unknown degradation products. The lower limit of quantification was established for AMB, CTZ, MP and PP. The developed RP-UPLC method was validated according to the International Conference on Harmonization (ICH) guidelines. This validated method is applied for simultaneous estimation of AMB, CTZ, MP and PP in commercially available syrup samples. Further, the method can be extended for estimation of AMB, CTZ, MP, PP and *levo*-cetirizine (LCTZ) in various commercially available dosage forms.

## Introduction

Ambroxol hydrochloride (AMB) is semi-synthetic derivative of vasicine obtained from Indian shrub *Adhatoda vasica*. It is a metabolic product of bromhexine. It is used as broncho secretolytic and an expectorant drug [1]. It simulates the transportation of the viscous secretions in the respiratory organs and reduces the stand stillness of the secretions. Cetirizine hydrochloride (CTZ) is an orally active and selective H_1_-receptor antagonist. It is piperazine derivative and metabolite of hydroxyzine. Combinations of AMB with CTZ in drug formulation used as antihistaminic H_1_ blockers. Methylparaben and propylparaben are used as either single or in combinations in drug products as antimicrobial preservatives to prevent alteration of product preparations. Methylparaben is the methyl ester of p-hydroxybenzoic acid and propylparaben is the propyl ester of *p*-hydroxybenzoic acid.

Liquid preparations are particularly susceptible to microbial growth because of the nature of their ingredients. Such preparations are protected by the addition of preservatives that prevent the alteration and degradation of the product formulation [2]. The finished product release specifications should include an identification test and a content determination test with acceptance criteria and limits for each antimicrobial preservative present in the formulation [3]. The finished product self-life specification should also include an identification test and limits for the antimicrobial preservatives present [3]. Hence their (MP and PP) antimicrobial and antifungal properties make them an integral part of the product formulation. This encourages the development of new stability indicating method for simultaneous estimation of all compounds (AMB, CTZ, MP and PP) to provide driving force in today’s pharmaceutical industry.

Several spectrophotometric methods have been reported for the qualitative and quantitative determination of AMB from pharmaceuticals formulations [4–7]. Various HPLC [8–11], GLC [12, 13], sequential injection technique coupled with monolithic column [14] LC-MS [15], capillary electrophoretic [16] and by capillary electrophoresis and fluorescence detection [17] are also reported for its determination from biological fluids. Literature survey revealed that several spectrophotometric [18–20] methods, HPLC methods [21–24], HPLC coupled to tandem mass spectroscopy [25], capillary electrophoretic [26, 27] have been also reported for determination of CTZ from Pharmaceutical formulations and biological fluids. Detailed literature survey for MP and PP revealed that many existing analytical procedures are available in literature for the determination of present preservatives studied, either alone or in combination with other drugs by HPLC and other techniques [11, 28–36].

A detailed literature survey for AMB + CTZ revealed that few analytical methods are available using spectrophotometric and HPLC where; Neela M. Bhatia *et al*. [37], describe RP-HPLC and spectrophotometric estimation of AMB and CTZ in combined dosage form; Mukesh Maithani *et al*. [38], simultaneous estimation of AMB and CTZ in tablet dosage form by RP-HPLC method; Trivedi Aditya *et al*. [39], development of modified spectrophotometric and HPLC method for simultaneous estimation of AMB and CTZ in tablet dosage forms; A. S. Birajdar *et al*. [40], simultaneous analysis of AMB with CTZ and of AMB with *levo*-Cetirizine dihydrochloride in solid dosage forms by RP-HPLC; NM Gowekar *et al*. [41], spectrophotometric estimation of AMB and CTZ from tablet dosage form. HPTLC method is also reported by S.B. Bagade *et al*. [42].

UPLC is a new category of separation technique based upon well-established principles of liquid chromatography, which utilizes sub-2 μm particles for stationary phase. These particles operate at elevated mobile phase linear velocities to affect dramatic increase in resolution, sensitivity and speed of analysis. Owing to its speed and sensitivity, this technique is gaining considerable attention in recent years for pharmaceuticals and biomedical analysis. In the present work, this technology has been applied to the method development and validation study of assay determination (AMB, CTZ, MP and PP) in liquid pharmaceutical formulation.

The combination of AMB and CTZ is not official in any pharmacopoeia. So far, no RP-UPLC stability indicating method has been reported for the rapid simultaneous determination of AMB, CTZ, MP and PP in liquid pharmaceutical formulation. Therefore, it is necessary to develop a new rapid and stability-indicating method for simultaneous determination of four compounds (AMB, CTZ, MP and PP) in liquid pharmaceutical formulation. The proposed method is able to separates AMB, CTZ, MP and PP with each other and from its all twelve (AMB impurities A, B, C, D, E and CTZ impurities A, B, C, D, E, F, CDH1) known impurities/degradation products and one unknown degradation product within 3.5 min. Thereafter, this method was validated according to the ICH guideline [43] and successfully applied for separation and quantification of all compounds of interest in the liquid and solid pharmaceutical formulation.

Ambroxol Impurity-A, B, C, D and E are official in British Pharmacopoeia [44]. Cetirizine specified impurities A, B, C, D, E and F are also official in British Pharmacopoeia [45]. Cetirizine CDH1 (impurity G as per British Pharmacopoeia) impurity is completely characterized in house (Dr. Reddy’s Laboratory) by using IR, Mass and NMR.

## Results and Discussion

### Method development and optimization

The main objective of the RP-UPLC method development was to rapid and simultaneous determination of AMB, CTZ, MP and PP in liquid pharmaceutical formulation were: the method should be able to determine assay of four compounds in single run and should be accurate, reproducible, robust, stability indicating, filter compatible, linear, free of interference from blank / placebo / impurities / degradation products and straightforward enough for routine use in quality control laboratory.

The spiked solution of AMB (120 μg/mL), CTZ (20 μg/mL), MP (40 μg/mL) and PP (4 μg/mL) was subjected to separation by RP-UPLC. Labeled claim of compounds and its working concentration is presented in Table 1. Initially the separation of all compounds was studied using water as a solvent-A and acetonitrile as a solvent-B on UPLC column (Eclipse Plus C18, RRHD, 50 × 2.1 mm; 1.8 μm) and Waters (UPLC) system with the linear gradient program. The flow rate of 0.5 mL/min was selected with regards to the backpressure and analysis time as well. During this study column oven temperature was capped at 50°C. When study performed with above condition we observed broad peak of all the compounds. Various types of solvent-A and B were studied to optimize the method, which were summarized in Table 3 with the observation. Based on above solvent selection study optimized UPLC parameters were; flow rate 0.5 mL/min; column oven temperature 50°C; gradient solvent program as per Table 2; 0.01M phosphate buffer in 0.1% triethylamine as a solvent-A and acetonitrile as a solvent-B.

**Fig. 1.**
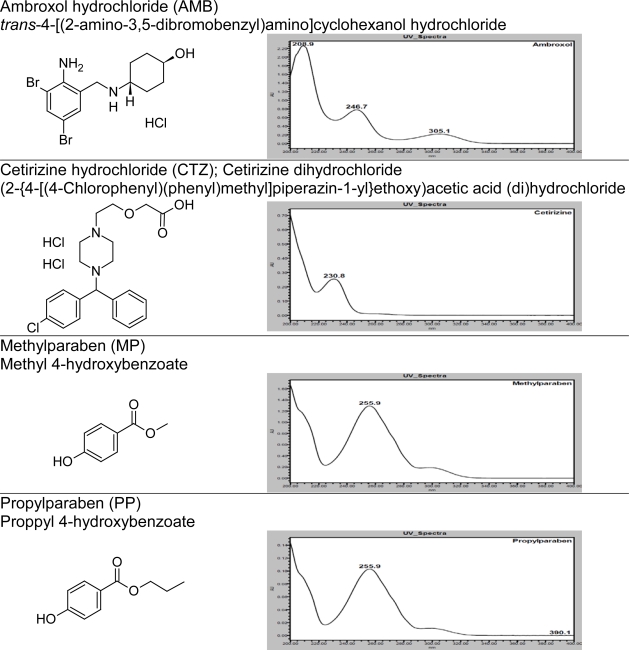
Chemical structures, UV spectrums and IUPAC name of AMB, CTZ, MP and PP

**Fig. 2.**
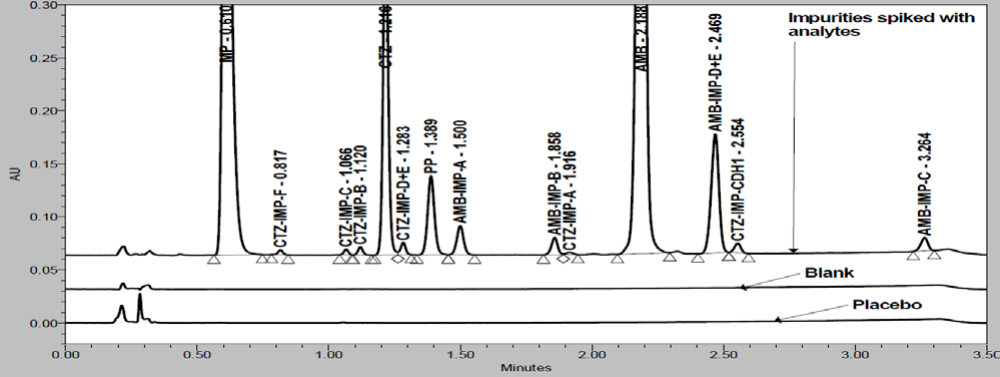
Overlay chromatograms of placebo, blank and spiked impurities along with analytes

**Fig. 3.**
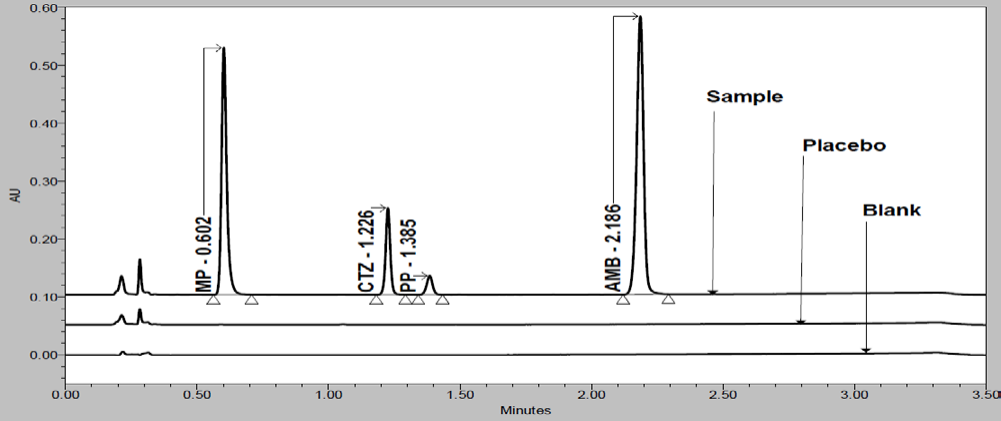
Overlay chromatograms of blank, placebo and sample preparation

In order to achieve symmetrical peak of all substances and more resolution between CTZ and PP different stationary phases were explored. Peak merging (CTZ and PP) was observed with Acquity BEH C8 (50 × 2.1 mm, 1.7μm) column. Poor resolution (R_S_=2.3 between CTZ and PP) was observed with Acquity BEH C18 (50 × 2.1 mm, 1.7μm) column. Finally desired separation with symmetrical peaks was obtained using Eclipse Plus C18, RRHD (50 × 2.1mm, 1.8μm) column. Column oven temperature is also studied (at low temperature and 50°C) and found that 50°C is more appropriate with respect to separation and peak shape. Based on compounds UV spectrums 237nm was found more appropriate for the simultaneous determination. Chemical structures, UV spectrums and IUPAC name of AMB, CTZ, MP and PP are presented in Figure 1. AMB, CTZ, MP and PP are well resolved with each other and also well resolved with all twelve known impurities/degradation products in reasonable time of 3.5 minutes which is presented in Figure 2. There was no any chromatography interference due to blank (diluent) and excipients (placebo) at the retention time of AMB, CTZ, MP and PP which was presented in Figure 3.

### Analytical parameters and validation

After satisfactory development of method it was subjected to method validation as per ICH guideline [43]. The method was validated to demonstrate that it is suitable for its intended purpose by the standard procedure to evaluate adequate validation characteristics (system suitability, accuracy, precision, linearity, robustness, solution stability, filter compatibility and stability indicating capability).

### Specificity

Specificity is the ability of the method to measure the analyte response in the presence of its potential impurities [43]. Forced degradation studies were performed to demonstrate selectivity and stability indicating capability of the proposed RP-UPLC method. Figure 2 and 3 are shows that there is no any interferences at the RT (retention time) of AMB, CTZ, MP and PP due to blank, placebo, impurities and degradation products.

**Fig. 4.**
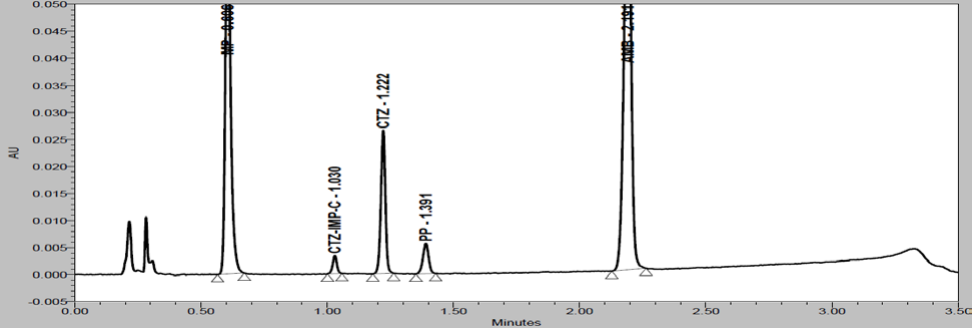
Chromatogram of acid degraded drug product

**Fig. 5.**
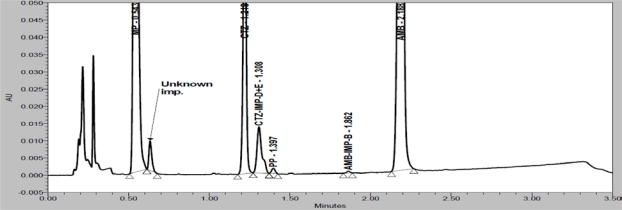
Chromatogram of base degraded drug product

**Fig. 6.**
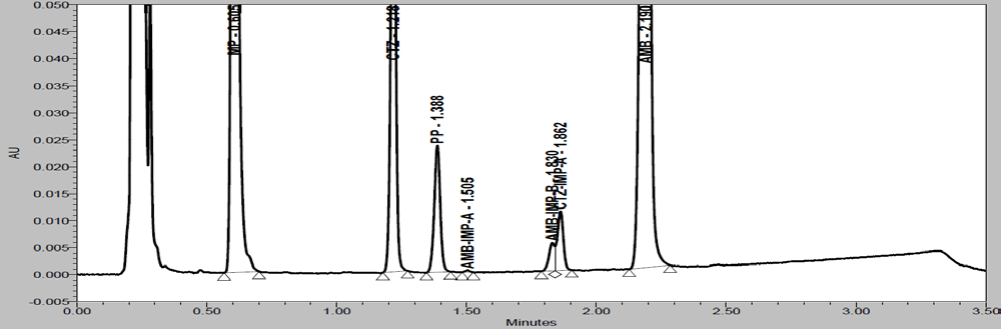
Chromatogram of peroxide degraded drug product

**Fig. 7.**
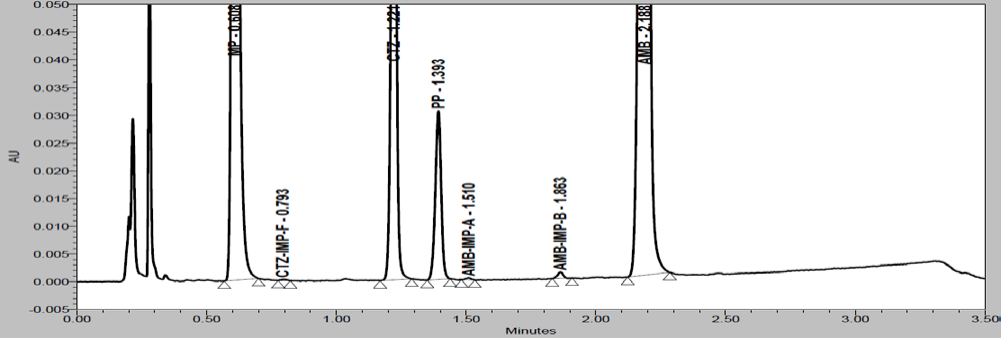
Chromatogram of heat degraded drug product

**Fig. 8.**
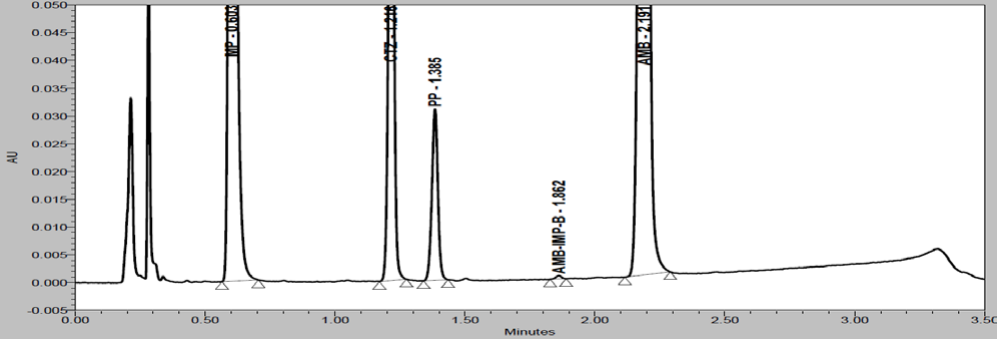
Chromatogram of photolytic degraded drug product

Degradation were observed when the drug product was subjected to acid hydrolysis (0.1N HCl, 60°C, 1h, Figure 4), base hydrolysis (0.1N NaOH, 60°C, 1h, Figure 5), oxidative (6% H_2_O_2_, 60°C, 1h, Figure 6), thermal (60°C, 1h, Figure 7) and photolytic degradation (1.2 million Lux hours, Figure 8). Significant degradation was observed when the drug product was subjected to base hydrolysis leading to the formation of unknown impurity after the peak of MP, CTZ impurity-D, E and AMB impurity-B (Figure 5). Peaks due to AMB, CTZ, MP and PP were investigated for spectral purity in the chromatogram of all exposed samples and found spectrally pure.

### Precision

#### Instrument precision: (Suitability of system)

System suitability parameters were measured so as to verify the system performance. System precision was determined on six replicate injections of standard preparation (Table 1). All important characteristics including % RSD, resolution (between CTZ and PP), tailing factor and theoretical plate number were measured. The percentage RSD of area counts of six replicate injections was below 1.0 %, which indicates that the system is precise. The results obtained are shown in Table 4. The parameters all complied with the acceptance criteria and system suitability was established.

#### Method precision: (Repeatability)

The precision of the assay method was evaluated by carrying out six independent determination of AMB, CTZ, MP and PP (120 μg/mL of AMB, 20 μg/mL of CTZ, 40 μg/mL of MP and 4 μg/mL of PP) test samples against qualified working standard. The method precision study shows the repeatability of the results obtained by the testing method. The % RSD (n=6) was 0.3 % for AMB, 0.5 % for CTZ, 0.4 % for MP and 0.7 % for PP, which are well within the acceptable limit of 2.0%. It was confirmed from results that the method is precise for the intended purpose (Table 5).

#### Intermediate precision: (Reproducibility)

The purpose of this study is to demonstrate the reliability of the test results with variations. The reproducibility was checked by analyzing the samples by different analyst using different chromatographic system and column on different day. The analysis was conducted in the same manner as the method precision and the % RSD of all six sets of sample preparations was determined (Table 5). The % RSD was 0.4 % for AMB, 0.6 % for CTZ, 0.7 % for MP and 0.9 % for PP, which are well within the acceptance criteria of 2.0%, so this study proved that the method to be rugged enough for day to day use.

### Accuracy

The accuracy of an analytical method is the closeness of test results obtained by that method compared with the true values. To confirm the accuracy of the proposed method, recovery experiments were carried out by standard addition technique. The accuracy of the method was carried out by adding known amounts of each drug corresponding to three concentration levels; 50, 100, and 150% of the label claim (Table 1) along with the excipients in triplicate. The samples were given the same treatment as described in sample preparation. The percentage recoveries of AMB, CTZ, MP and PP at each level and each replicate were determined. The mean of percentage recoveries (n=3) and the relative standard deviation was calculated. The amount recovered was within ± 1.0 % of amount added, which indicates that there is no interference due to excipients present in liquid oral formulation. It was confirmed from results that the method is highly accurate (Table 6).

### Linearity

The linearity of an analytical method is its ability to elicit test results that are directly, or by a well-defined mathematical transformation, proportional to the concentration of analyte in sample within a given range. The nominal concentrations of standard and test solutions for AMB, CTZ, MP and PP were 120, 20, 40 and 4 μg/mL, respectively. The response function was determined by preparing standard solutions at seven different concentration levels ranging from 30.04–240.32 μg/mL for AMB, 5.01–40.08 μg/mL for CTZ, 9.97–79.76 μg/mL for MP and 1.005–8.04 μg/mL for PP (25 to 200% of analyte concentration). The response was found linear from 25% to 200% of standard concentration. For all compounds the correlation coefficient was greater than 0.999. The regression statistics are shown in Table 7.

### Lower limit of quantification (LLOQ)

The signal-to-noise ratio (*S/N*) method was adopted for the determination of lower limit of quantification. The lower limit of quantification is estimated as ten times the *S/N* ratio. Quantification limit was achieved by injecting a series of possible dilute solutions of AMB, CTZ, MP, and PP. The precision was also established at quantification level. The % RSD of peak area was well within the acceptance limit of not more than 10 %. The determined lower limit of qualification and precision at LLOQ values for AMB, CTZ, MP and PP are presented in Table 8.

### Robustness

The robustness of an analytical procedure is a measure of its capacity to remain unaffected by small, but deliberate variations in method parameters and provides an indication of its reliability during normal usage. The effect of change in flow rate (± 0.05 mL/min) and column oven temperature (± 5°C) on the retention time, resolution (between CTZ and PP), theoretical plates and tailing factor were studied. During study other chromatographic conditions were kept same as per the experimental section. It was conformed from results that the method is robust with respect to variability in above conditions (Table 9).

### Stability of sample in diluent

Drug stability in pharmaceutical formulations is a function of storage conditions and chemical properties of the drug, preservative and its impurities. Condition used in stability experiments should reflect situations likely to be encountered during actual sample handling and analysis. Stability data is required to show that the concentration and purity of analyte in the sample at the time of analysis corresponds to the concentration and purity of analyte at the time of sampling. Stability of sample solution was established by storage of sample solution at ambient temperature (25°C) for 24h. Sample solution was re-analyzed after 12 and 24h time intervals and assay were determined for the compounds (AMB, CTZ, MP and PP) and compared against fresh sample. Sample solution did not show any appreciable change in assay value when stored at ambient temperature up to 24h, which are presented in Table 10. The results from solution stability experiments confirmed that sample solution was stable for up to 24h during assay determination.

### Filter compatibility

Filter compatibility was performed for nylon 0.22 μm syringe filter (Pall Life sciences) and PVDF 0.22 μm syringe filter (Millipore). To confirm the filter compatibility in proposed analytical method, filtration recovery experiment was carried out by sample filtration technique. Sample was filtered through both syringe filters and percentage assay was determined and compared against centrifuged sample. Sample solution was not showing any significant changes in assay percentage with respect to centrifuged sample. Percentage assay results are presented in Table 11. In displayed result difference in % assay was not observed more than ±0.5, which indicates that both syringe filters having a good compatibility with sample solution.

### Application of the method to dosage forms

The present method was applied for the estimation of drugs and preservatives in the commercially available various dosage forms. The results obtained are as shown in Table 12. Based on obtained results developed method is suitable for the various marketed dosage forms. Developed method also proved the suitability for preservatives determination in various liquid dosage forms.

## Experimental

### Materials and Reagents

Drug product, placebo solution, working standards and reference standards were provided by Dr. Reddy’s laboratories Ltd., Hyderabad, India. HPLC grade acetonitrile and methanol were obtained from J.T.Baker (NJ., USA). GR grade potassium dihydrogen phosphate, GR grade orhtophosphoric acid and GR grade triethylamine were obtained from Merck Ltd. (Mumbai, India). 0.22 μm nylon membrane filter and nylon syringe filters were purchased from Pall life science limited (India). 0.22 μm PVDF syringe filter was purchased from Millipore (India). High purity water was generated by using Milli-Q Plus water purification system (Millipore^®^, Milford, MA, USA).

### Equipments

Cintex digital water bath was used for specificity study. Photo stability studies were carried out in a photo-stability chamber (SUNTEST XLS+, ATLAS, Germany). Thermal stability studies were performed in a dry air oven (Cintex, Mumbai, India).

### Chromatographic conditions

Analyses were performed on Acquity UPLC^™^ system (Waters, Milford, USA), consisting of a binary solvent manager, sample manager and PDA (photo diode array) detector. System control, data collection and data processing were accomplished using Waters Empower^™^-2 chromatography data software. The chromatographic condition was optimized using Agilent Eclipse Plus C18, RRHD 1.8 μm (50 mm × 2.1 mm) column. Mixture of 0.01M phosphate buffer (KH_2_PO_4_) in 0.1% triethylamine was used as a solvent-A and acetonitrile was used as solvent-B. Solvents-A and B was filtered through 0.22 μm nylon membrane filter and degassed under vacuum prior to use. The separation of AMB, CTZ, MP, PP and all impurities was achieved by gradient elution using solvent-A and B (Table 2). Mixture of water and methanol in the ratio of 50:50 (v/v) respectively was used as a diluent. The finally selected and optimized conditions were as follows: injection volume 4 μL, gradient elution (Table 2), at a flow rate of 0.5 mL/min at 50°C (column oven) temperature, detection wavelength 237 nm. Under these conditions, the backpressure in the system was about 6,000 psi. The stress degraded samples and the solution stability samples were analyzed using a PDA detector covering the range of 200–400nm.

### Standard solution preparation

Standard solution was prepared by dissolving standard substances in diluent to obtain solution containing 120 μg/mL of Ambroxol hydrochloride, 20 μg/mL of Cetirizine hydrochloride, 40 μg/mL of Methylparaben and 4 μg/mL of Propylparaben.

### Sample solution preparation

An accurately weighed 2 gm of sample solution was taken into the 100 mL volumetric flask. About 70 mL of diluent was added to this volumetric flask and sonicated in an ultrasonic bath for 5 min. This solution was then diluted up to the mark with diluent and mixed well. It was then filtered through 0.22 μm PVDF syringe filter and the filtrate was collected after discarding first few milliliters.

### Placebo (other substances without AMB, CTZ, MP and PP) solution preparation

An accurately weighed 2 gm of placebo solution was taken into the 100 mL volumetric flask. About 70 mL of diluent was added to this volumetric flask and sonicated in an ultrasonic bath for 5 min. This solution was then diluted up to the mark with diluent and mixed well. It was then filtered through 0.22 μm PVDF syringe filter and the filtrate was collected after discarding first few milliliters.

### Market product sample solution preparation (for oral solution)

An accurately weighed X gm of sample solution was taken into 100 mL volumetric flask (where X= 4 gm for Xyzal^®^ [UCB, India Pvt. Ltd.; B.No.-VO 10001], 2 gm of ZyrCold^®^ [UCB, India Pvt. Ltd.; B.No.-LI10035] and 2 gm of Relent^®^ [Dr. Reddy’s Lab. Ltd. India; B.No.-L 0590]). About 70 mL of diluent was added to this volumetric flask and sonicated in an ultrasonic bath for 3 min. This solution was then diluted up to the mark with diluent and mixed well. It was then filtered through 0.22 μm PVDF syringe filter and the filtrate was collected after discarding first few milliliters.

### Market product sample solution preparation (for oral tablet)

Twenty tablets were crushed to fine powder. An accurately weighed portion of the powder equivalent to 5 mg of CTZ was taken into 100 mL volumetric flask (Cetzine^®^ Tablets [GSK Pharmaceuticals Ltd.; B.No.-L473], Dio-1^®^ Tablets [Unison pharmaceuticals; B.No.-2005] and ZyrCold^®^ Tablets [UCB, India Pvt. Ltd.; B.No.-5829]). About 70 mL of diluent was added to this volumetric flask and sonicated in an ultrasonic bath for 15 min. This solution was then diluted up to the mark with diluent and mixed well. It was then filtered through 0.22 μm PVDF syringe filter and the filtrate was collected after discarding first few milliliters.

## Conclusion

A gradient RP-UPLC method was successfully developed for the simultaneous estimation of AMB, CTZ, MP and PP in liquid pharmaceutical formulation. The developed method is selective, precise, accurate, linear, filter compatible and robust. Forced degradation data proved that the method is specific for the analytes and free from the interference of placebo / known impurities / degradation products and unknown degradation products. The run time (3.5 min) enables for rapid determination of drugs and preservatives. Moreover, it may be applied for individual and simultaneous determination of AMB, CTZ, LCTZ, MP and PP compound in pharmaceutical drug product and substance. Also it can be utilized for determination of assay, blend uniformity and content uniformity of pharmaceutical products (CTZ tablets and AMB+CTZ tablets), where sample load is higher and high throughput is essential for faster delivery of results.

## Figures and Tables

**Tab. 1.** Formulation label claim with its working concentration (specification limit)

**Compd.**	**Formulation label claim per 5 mL**	**Working concentration**	
		**mg/mL**	**μg/mL**
AMB	Ambroxol hydrochloride 30 mg	0.12	120
CTZ	Cetirizine hydrochloride 5 mg	0.02	20
MP	Methylparaben 10 mg	0.04	40
PP	Propylparaben 1 mg	0.004	4

**Tab. 2.** **Gradients program for elution**

**Time (min)**	**Flow rate (mL/min)**	**% Solvent-A**	**% Solvent-B**	**Curve**
Initial	0.5	70	30	Isocratic
0.2	0.5	70	30	Isocratic
3.0	0.5	5	95	Linear
3.1	0.5	70	30	Isocratic
3.5	0.5	70	30	Equilibration

**Tab. 3.** Summary of solvent used to optimize the method

**Solvent-A**	**Solvent-B**	**Observation**	
		**Retention time (t_R_)**	**USP tailing**
Water	Acetonitrile	AMB=0.947; MP=1.373	AMB=2.8; MP=2.0
		CTZ=2.016; PP=2.748	CTZ=2.5; PP=1.5
0.1M KH_2_PO_4_	Acetonitrile	AMB=1.101; MP=1.451	AMB=1.8; MP=1.4
		CTZ=2.234; PP=2.851	CTZ=1.3; PP=1.3
0.1M KH_2_PO_4_ buffer (pH 3.0 with H_3_PO_4_)	Acetonitrile	AMB=1.223; MP=1.477	AMB=1.7; MP=1.4
		CTZ=2.019; PP=2.835	CTZ=1.3; PP=1.2
0.01M KH_2_PO_4_ + 0.1% triethylamine	Acetonitrile	AMB=2.185; MP=0.605	AMB=0.9; MP=1.3
		CTZ=1.217; PP=1.389	CTZ=1.0; PP=1.0

USP = United state pharmacopoeia

**Tab. 4.** System suitability results (precision and intermediate precision)

**Test**	**Parameters**	**MP**	**CTZ**	**PP**	**AMB**	**Proposed criteria**
Precision (n=6)	USP resolution	–	–	4.34	-–	NLT 3.5
	USP tailing	1.3	1.0	1.0	0.9	NMT 1.5
	USP plate count	4549	24009	18630	33643	NLT 3000
	Area % RSD	0.2	0.2	0.4	0.1	NMT 2.0%

Intermediate precision (n=6)	USP resolution	–	–	4.35	–	NLT 3.5
	USP tailing	1.3	1.0	1.0	0.9	NMT 1.5
	USP plate count	4656	23951	18444	33537	NLT 3000
	Area % RSD	0.3	0.2	0.5	0.2	NMT 2.0%

NLT= Not less than; NMT= Not more than.

**Tab. 5.** Precision (n=6) and Intermediate precision (n=6) results

**Substance**	**Precision at 100%**		**Intermediate precision**	

	**Mean % assay**	**% RSD**	**Mean % assay**	**% RSD**
AMB	101.1	0.3	101.0	0.4
CTZ	99.3	0.5	99.5	0.6
MP	98.1	0.4	98.3	0.7
PP	97.5	0.7	97.2	0.9

**Tab. 6.** Accuracy results

**Compd.**	**At 50% (n=3)**		**At 100% (n=3)**		**At 150% (n=3)**	

	**%Recovery**	**%RSD**	**%Recovery**	**%RSD**	**%Recovery**	**%RSD**

AMB	100.1	0.3	99.8	0.2	99.8	0.2
CTZ	99.7	0.4	99.9	0.3	100.2	0.2
MP	100.2	0.3	99.8	0.2	99.7	0.2
PP	100.6	0.5	100.4	0.4	99.6	0.6

**Tab. 7.** Regression statistics

**Compd.**	**Linearity range (μg/mL)**	**Correlation coefficient (r^2^)**	**Linearity (Equation)**	**Y-intercept bias in %**
AMB	30.04 to 240.32	0.9996	y = 9020.7497(x) + 6130.5328	0.557
CTZ	5.01 to 40.08	0.9998	y = 11111.1220(x) – 263.1311	−0.117
MP	9.97 to 79.76	0.9997	y = 18542.8499(x) + 5084.6885	0.676
PP	1.005 to 8.04	0.9997	y = 16185.3682(x) − 118.5082	−0.181

**Tab. 8.** LLOQ and precision at LLOQ (n=6) results

	**MP**	**CTZ**	**PP**	**AMB**
LLOQ (μg/mL)	0.12	0.18	0.13	0.16
Precision (% RSD)	3.5	4.3	5.7	4.8

**Tab. 9.** Robustness study results

**Condition**	**Parameters**	**MP**	**CTZ**	**PP**	**AMB**	**Proposed criteria**
Normal methodology	USP resolution	–	–	4.34	–	NLT 3.5
	USP tailing	1.3	1.0	1.0	0.9	NMT 1.5
	USP plate count	4549	24009	18630	33643	NLT 3000
	Retention time in min	0.603	1.224	1.385	2.183	–

At flow rate 0.45 mL/min	USP resolution	–	–	4.79	–	NLT 3.5
	USP tailing	1.3	1.0	1.0	0.9	NMT 1.5
	USP plate count	4657	24838	18993	34634	NLT 3000
	Retention time in min	0.667	1.309	1.498	2.319	–

At flow rate 0.55 mL/min	USP resolution	–	–	3.88	–	NLT 3.5
	USP tailing	1.3	1.0	1.0	0.9	NMT 1.5
	USP plate count	4790	22386	17442	32067	NLT 3000
	Retention time in min	0.549	1.160	1.300	2.081	–

At 45°C column oven temp.	USP resolution	–	–	5.3	–	NLT 3.5
	USP tailing	1.3	1.0	1.0	0.9	NMT 1.5
	USP plate count	5310	23455	19314	32483	NLT 3000
	Retention time in min	0.628	1.224	1.434	2.186	–

At 55°C column oven temp.	USP resolution	–	–	3.6	–	NLT 3.5
	USP tailing	1.3	1.0	1.0	0.9	NMT 1.5
	USP plate count	4481	22939	16790	32440	NLT 3000
	Retention time in min	0.580	1.210	1.337	2.172	–

**Tab. 10.** Solution stability results

**Time intervals**	**AMB**	**CTZ**	**MP**	**PP**
% Assay Initial	100.7	99.5	98.0	97.6
% Assay after 12h	100.3	99.6	98.2	97.7
% Assay after 24h	100.4	99.3	98.1	97.4

**Tab. 11.** Filter compatibility results (Assay % w/w)

**Compd.**	**Centrifuged**	**PVDF filter 0.22μm (Millipore)**	**Nylon filter 0.22μm (Pall Life Sciences)**
AMB	100.5	100.3	100.3
CTZ	99.7	99.5	99.4
MP	98.3	98.3	98.5
PP	97.5	97.6	97.3

**Tab. 12.** Results of market products (mg/ 5 mL for syrup and mg/ tablets for tablets)

**Product Name and Labeled claim (in mg)**	**AMB**	**CTZ**	**MP**	**PP**	**LCTZ**
ZyrCold Syrup [AMB(30); CTZ(2.5)]	29.8	2.47	9.53	0.94	N.A.
Relent Syrup [AMB(30); CTZ(2.5)]	30.1	2.48	10.10	1.01	N.A.
ZyrCold Tablets [AMB(30); CTZ(2.5)]	29.7	2.46	N.A.	N.A.	N.A.
Cetzine Tablets [CTZ(10)]	N.A.	9.93	N.A.	N.A.	N.A.
DOI-1 Tablets [CTZ(10)]	N.A.	9.72	N.A.	N.A.	N.A.
Xyzal Syrup [LCTZ (2.5)]	N.A.	N.A.	10.5	1.07	2.51

N.A. not applicable
